# Fetal Growth and Osteogenesis Dynamics during Early Development in the Ovine Species

**DOI:** 10.3390/ani13050773

**Published:** 2023-02-21

**Authors:** Sara Succu, Efisiangelo Contu, Daniela Bebbere, Sergio Domenico Gadau, Laura Falchi, Stefano Mario Nieddu, Sergio Ledda

**Affiliations:** 1 Department of Veterinary Medicine, University of Sassari, 07100 Sassari, Italy; 2 Istituto Zooprofilattico Sperimentale della Sardegna, 07100 Sassari, Italy

**Keywords:** prenatal growth, osteogenesis dynamics, ultrasound, sheep

## Abstract

**Simple Summary:**

Our study represents an innovative attempt to describe fetal skeleton development during the first part of gestation in sheep using ultrasonography associated with a differential staining technique that allows the observation of the first ossification processes. The timing of ossification and the growth rate of skeletal components are very important to assess normal prenatal growth. This knowledge may be useful for gestational management and early diagnosis of skeletal diseases, especially in the early period of pregnancy when bone growth is very fast in the ovine species.

**Abstract:**

Increased knowledge of the developmental processes during gestation could provide valuable information on potential alterations in embryonic/fetal development. We examined the development of ovine conceptus between the 20th and 70th day of gestation with three convergent analyses: (1) uterus ultrasound examination and measurement (eco) of crown–rump length (CRL) and biparietal diameter (BPD) of the conceptus; (2) direct measurement (vivo) of CRL and BPD of the conceptus outside the uterus (3) osteo–cartilage dynamics during development by differential staining. No significant differences were observed between eco and vivo measurements for CRL and BPD in all examined concepti. CRL and BPD, instead, showed a significant positive linear correlation with gestational age. The study of osteogenesis dynamics has demonstrated a completely cartilaginous ovine fetus at up to 35 days of gestation. The ossification begins in the skull (40th day) and is almost complete between the 65th and the 70th of pregnancy. Our study highlighted that CRL and BPD are accurate parameters for gestational age estimation in the first part of sheep pregnancy and provides an overview of osteochondral temporal dynamics. Furthermore, tibia ossification is a valid parameter to estimate fetal age by ultrasound.

## 1. Introduction

Since the beginning of studies on reproduction in animal and human models, the knowledge of the mechanisms and the description of the main events related to gestation have represented an intense field of study and research. Gestation in mammalians is a complex phenomenon and any physiological changes that occur during fetal development can affect the subsequent health and well-being into adulthood. Prenatal development is influenced by several factors, such as genetic background, size and age of the mother, size of the offspring, determination of the sex and function of the placenta. On the other hand, many pathological conditions or changes in homeostasis such as metabolic disorders, infectious events, stress and temperature could negatively affect the growth rate of embryonic or fetal development [1,2].

The first part of pregnancy is considered particularly sensitive because it concerns related physiological processes, such as the development of the corpus luteum (as a temporary endocrine reservoir), the implantation of the conceptus in the uterus, its growth, and the development of an efficient functional placenta [3,4].

Increased knowledge of the developmental processes during gestation could provide valuable information on potential alterations in embryonic/fetal development. Sheep have been largely used as an experimental model to study prenatal growth during gestation [5]. Indeed, ovine conceptus development closely resembles the human one in terms of weight and number of births [6,7]. Furthermore, sheep have a good tolerance to uterus manipulation and its pregnancy is considered a useful model for the study of skeletal development, because the bone tissue is very similar to human in terms of density and strength [8]. Bones represent the framework of support and protection of the body and are considered the passive organs of movement [9]. In this way, the fetal sheep can also be used as a practical model to study the skeletal system development during the uterine period [10].

Ultrasonography is an advanced technique that provides greater quality images and is useful to identify and to study embryonic and fetal anatomical structures. The ultrasonographic assessment of skeletal growth represents an important marker to evaluate the correct development and health of the conceptus and to determine the gestational age [11,12]. Despite being a well-validated technique, the reliability of ultrasonography can be affected by the quality of the ultrasonic equipment, the technique for obtaining the images, the frequency of examinations, the evaluation period, and the experience of the operator [13].

Furthermore, sheep represents a useful and low-cost model to study dynamic fetal growth due to the possibility of finding fetuses of different gestational age from regularly slaughtered pregnant animals. In these samples, a precise ultrasound fetal growth can be performed, combined with a direct measurement of the concepti and evaluation of skeletal development using specific staining.

For this purpose, we have examined the development of the ovine conceptus between the 20th and 70th day of gestation in regularly slaughtered ewes. Conceptus growth was measured by ultrasound of the pregnant uterus and then by direct measurement of the conceptus after removal from the uterus. The development of the skeletal system and the dynamics of ossification were studied by clarification and differential cartilage-bone staining of the conceptus.

## 2. Materials and Methods

All chemicals in this study were purchased from Sigma Chemical CO (St. Louis, MO, USA) unless stated otherwise.

### 2.1. Conceptus Collection and Measurements

A total of 70 concepti of different gestational ages were obtained at local slaughterhouses from pregnant female Sarda sheep. These pregnant females were regularly slaughtered in accordance with European Regulations (Regulation of European Parliament and of the Council 853/2004; 627/2019; 1/2005; 1255/97) and the pregnant uteri were recovered after opening the abdominal cavity. The uteri were subjected to ultrasound using a Philips HD 11 and a 5–8 MHz micro convex probe. The probe was positioned on the ventral side of the pregnant uterus and the conceptus was carefully measured. According to [12], the morphometric parameters crown–rump length (CRL) and bi-parietal diameter (BPD) were measured in each conceptus and used to date the gestational age [14]. After ultrasound examination, each conceptus was recovered by an incision along the uterine body and both horns. The measurement of CRL was recorded directly by a metal ruler and the conceptus was processed for differential staining as described below.

### 2.2. Conceptus Staining for the Study of Osteogenesis Dynamics

The development of the skeletal system and the dynamics of ossification were assessed by a selective staining for bone and cartilage, according to [15], with some modifications. In brief, specimens were prepared by removing skin and organs, especially liver, intestine, kidneys and were then dehydrated and fixed in 95% ethanol for at least 7 days. It was very important to use ethanol as a fixative as well as other fixatives such as formalin to avoid non-specific staining of embryonic structures. For further removal of fatty tissue and for tissue permeabilization, specimens were exposed to 100% acetone overnight at room temperature. Then, they were transferred to Alcian blue stain (Ab), specific for cartilage. In detail, the specimens were immersed in a dye solution containing 10 mg of Alcian blue dissolved in 4 mL of H_2_O MilliQ, 80 mL of absolute ethanol and 20 mL of (glacial) acetic acid, for three days. It is also important to specify that embryos with an age of less than 30 days cannot be processed due to the richness of glycosaminoglycans of the young connective tissue that interferes with the staining of the cartilage matrix.

After that, specimens were exposed to decreasing concentrations of ethanol (ethanol 70% for 3 h, ethanol 70% for 3 h, ethanol 40% for 3 h, ethanol 15% for 3 h) for rehydration and then transferred to 1% potassium hydroxide (KOH) for 2 days for clarification. Then the specimens were immersed in dye solution specific for bone tissue containing 1 mg of Alizarin red stain (Ar) in 100 mL of 1% KOH solution, for at least 3 days and this solution had to be changed daily. For concepti aged 45 days or more it was necessary to double the Ar concentration. After that, the specimens were exposed to 1% KOH for 12 h to overnight for hydrolysis of soft tissues, leading to transparency and allowing visualization of stained skeletal elements. Finally, the samples were transferred to 100% glycerol for long-term storage. After staining, the specimens were observed using a stereomicroscope with 60X magnification and photographed.

### 2.3. Statistical Analysis

The normality of data distribution was analyzed using a Kolmogorov–Smirnov test. A General Linear Model method (GLM) with Tukey pairwise comparisons were used to analyze the morphometric measurements (CRL and BPD) of each conceptus where: Y = μ + measurements (by ultrasound vs. in vivo) + time (days of pregnancy) + measurements x time. The measurements and time were considered as fixed factors. All results are expressed as mean ± S.E.M. Statistical correlation between each morphometric measurements and gestational age were investigated by using the Pearson’s correlation coefficient. A *p*-value ≤ 0.05 was considered the minimum level of significance. Data were analyzed using Statistical Software Minitab^®^ 18.1 (2017 Minitab).

## 3. Results

### 3.1. Concepti Measurements

**Figure 1:**
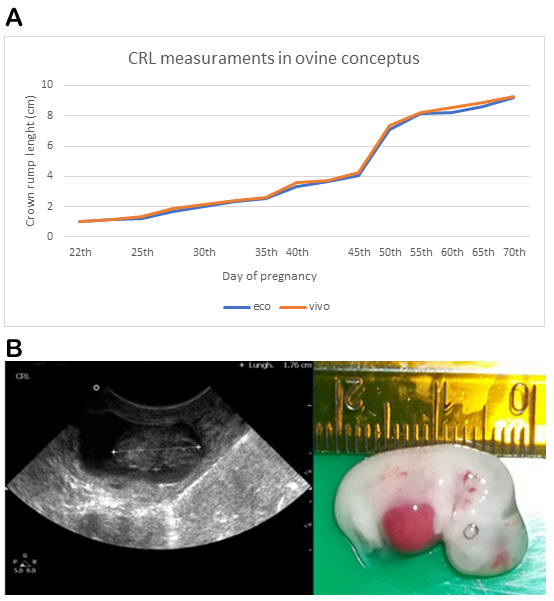
(**A**): crown rump length (CRL) in ovine conceptus from day 22 to day 70 of pregnancy measured by ultrasonography (eco) or directly (vivo) with a ruler. No significant difference was shown in the measurements obtained by the two different systems (eco vs. vivo). (**B**): Ultrasound measurement of CRL of a pregnant uterus at about 30 days of gestation (on the left) and the direct measurement of the same embryo (on the right).

As shown in Figure 1, the increase in CRL was constant and statistically significant from day 22 to day 45 of pregnancy in all measured concepti (*p* < 0.001).

No significant differences were observed between the measurements of the concepti performed by ultrasonography (eco) and by direct measurement (vivo), so the values of CRL obtained by the two different measurement methods are consistent (Figure 1 and Supplementary Data).

**Figure 2:**
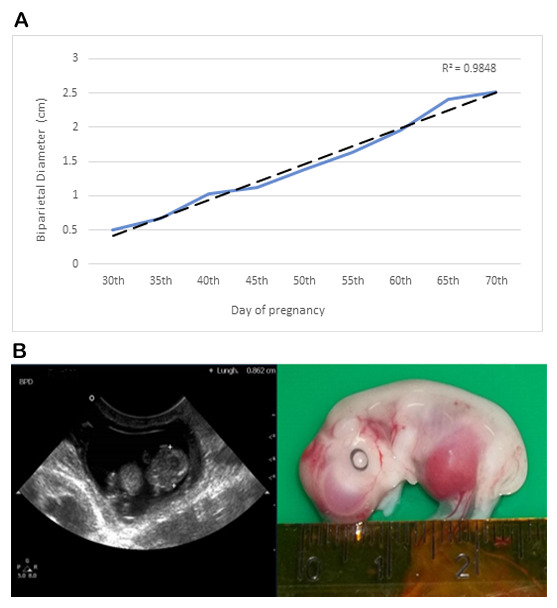
(**A**): Biparietal diameter (BPD) growth in ovine concepti from day 30 to day 70 of pregnancy measured by ultrasonography (blue continuous line) and linear correlation between BPD and gestational age (black dashed line; R^2^ = 0.98; *p* < 0.05). (**B**): Ultrasound measurement of BPD in a conceptus at 38 days of gestation.

Similarly, BPD growth (Figure 2 and Supplementary Data) was constant and statistically significant from day 30 to day 70 of pregnancy in all measured concepti (*p* < 0.001).

The two morphometric measurements were significantly (*p* < 0.05) correlated with the gestational age, showing a positive linear correlation with the increasing gestational age, and more specifically CRL eco R^2^ = 0.9729, CRL in vivo R^2^ = 0.9736 and BPD R^2^ = 0.9838.

### 3.2. Study of Osteogenesis Dynamics

**Figure 3:**
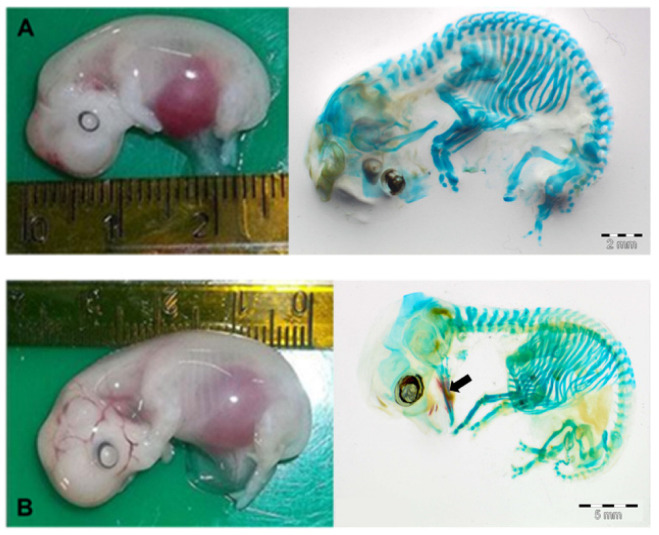
(**A**): ovine fetus at about 35 days of gestation (on the left). On the right, the same fetus stained with Alizarin red stain + Alcian blue stain. The skeleton is totally cartilaginous; indeed, it is colored blue. (**B**): ovine fetus at about 40 days of gestation (on the left). On the right, the same fetus stained with Alizarin red stain + Alcian blue stain. It is possible to observe the initial ossification of the mandible, in red (black arrow).

**Figure 4:**
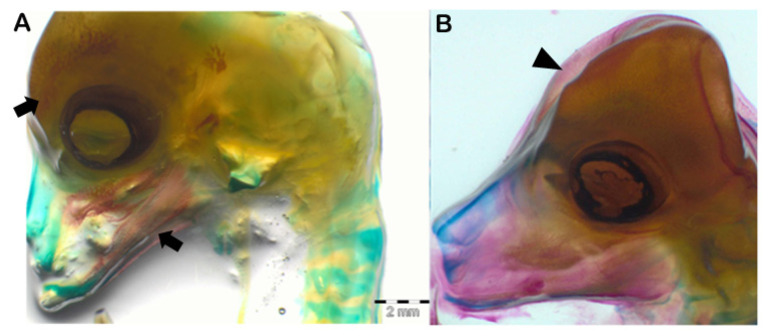
(**A**): ossification of the mandible and maxilla and extension of the ossification to the splanchnocranium (50 days), black arrows. (**B**): intra-membranous ossification of the neurocranium (approximately 60 days), black arrowhead.

We examined N = 20 concepti aged between 25 and 70 days of gestation, stained with the method described above. As shown in Figure 3A, the ovine fetus remained completely cartilaginous up to 35 days of gestation. The ossification began in the skull, starting from the mandible, towards the 40th day of pregnancy (Figure 3B), continued to the maxilla around the 45th day, then to the rest of the splanchnocranium between the 50th and the 60th day of gestation, and finally to the neurocranium between the 60th and the 70th days (Figure 4).

**Figure 5:**
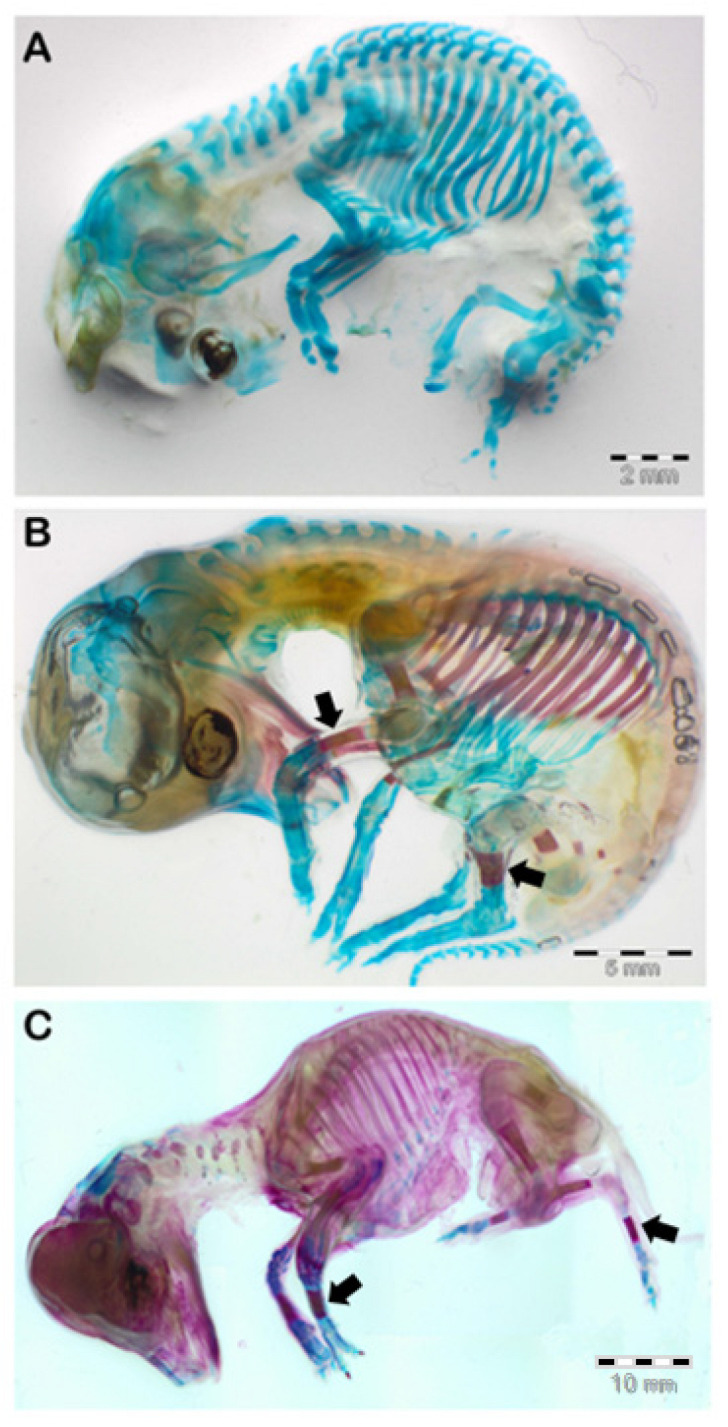
Progression of the ossification of the forelimbs and hindlimbs at 35 (**A**), 50 (**B**) and 65 (**C**) days of gestation. (**A**): The ovine fetus remained completely cartilaginous up to 35 days of gestation. (**B**): Ossification of radius, ulna and tibia is observed starting from the 50th day of gestation (black arrows). (**C**): progressive ossification of the metacarpal and metatarsal is noticeable at 65 days (black arrows).

The process of ossification of the limbs also began around the 40th day of gestation, with the appearance of ossification nuclei in the humerus and radius diaphysis. At 45 days, the process became more evident, the nuclei elongate and were also evident in the ulna and distal portion of the scapula. At 50 days, the diaphysis of the humerus, radius and ulna are ossified (Figure 5B). At 60 days, the ossification of the middle third of the metacarpal was observed (Figure 5C), while the remaining segments do not vary from the previous one. At 70 days of gestation, ossification nuclei appear in the phalanges.

The process of ossification of the hind limbs is observed starting from the 45th day of gestation, when small areas of ossification are visualized in the middle third of femur and tibia. From the 50th day of gestation, the tibia underwent considerable and rapid development, becoming the most developed bone segment of the entire body (Figure 5B). The elongation of this segment takes on a characteristic trapezoidal shape and remains linear between the 60th and the 70th day of gestation.

**Figure 6:**
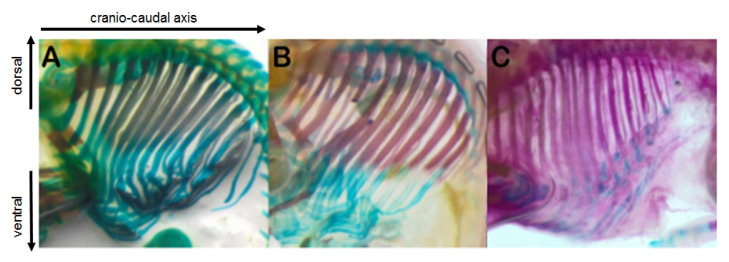
Ossification of the ribs at 45 (**A**), 50 (**B**) and 65 (**C**) days of gestation. The arrows indicate the cranio–caudal and the dorsal ventral axes of the conceptus in (**A**–**C**).

The ossification of the spine occurs after 50 days and begins from the thoracic, lumbar, and sacral portions. The cervical portion ossified at 60 days while the coccygeal one after 65 days. The ossification of the ribs is evident starting from approximately the 45th day of gestation in the dorsal portions (Figure 6A). It is more marked on the 50th day, always in the dorsal portion (Figure 6B), while between 65 and 70 days it extended to the ventral portion. The costochondral junctions and the sternal ribs remained cartilaginous (Figure 6C). The ossification of the sternum occurred later compared to the ribs. The centers of ossification appeared around the 65th day and at the 70th day of gestation the structure is yet not completely ossified.

Table 1 provides a summary of the dynamics of ossification during conceptus growth between day 30 and 70 of gestation in sheep.

## 4. Discussion

The present study described the development of the ovine conceptus by ultrasound analysis and the dynamics of ossification during the first 70 days of gestation.

Three convergent analyses were performed in different stages of gestation: (1) ultrasound examination of the uterus and measurement of crown–rump length (CRL) and biparietal diameter (BPD) of the conceptus; (2) direct measurement of CRL and BPD of the conceptus outside the uterus and (3) osteo–cartilage dynamics during development by differential staining. Ultrasonography is widely used in many domestic species to measure fetal development and to determine the gestational age [16,17]. In our study, sheep concepti ranging between 22 and 70 days of presumptive gestation were examined by ultrasound. In this time window, the most representative morphometric parameter was the CRL, due to the linearity of the shape of the embryo. Indeed, the CRL is considered one of the selection parameters to calculate the gestational age, especially in the first stages of pregnancy [18]. Our data are in accordance with the foregoing statements; we have highlighted a high correlation between CRL, and gestational age and no significant differences were detected between ultrasonography (eco) and direct (vivo) measurement (Figure 1). This result is very interesting as it demonstrates the reliability of ultrasound analysis for the determination of gestational stages in the ovine species at the beginning of pregnancy. Recently, several studies investigated the usefulness of advanced ultrasound approaches in the estimation of fetal age. Three-dimensional (3D) ultrasound nevertheless represents a valuable tool for measuring fetal volumes, providing precise assessment of fetal structures. Although widely used in human clinical obstetrics, it has had limited applications in veterinary reproduction. In the mare and the cow, 3D ultrasound has been tested for the acquisition of fetal measurements, sexing and predicting weight at birth [19,20]. However, the need to standardize acquisition procedures and the costs for the equipment may limit the application in clinical practice. Another promising approach could be represented by quantitative ultrasound. Aimed at investigating the interactions between ultrasound waves and tissue microstructure, quantitative ultrasound has been employed in humans to assess fetal lung maturity [21,22] and bone mineralization dynamics [23]. In the ovine, quantitative ultrasound has been employed to investigate morphophysiological changes in maternal–fetal tissues such as placentomes, lungs, liver and kidneys during pregnancy [24] paving the way to future applications in farm animals.

As already described [12] in the ovine species, during clinical routines the CRL measurement by ultrasound is less reliable after the 40th day of pregnancy, as a consequence of rapid fetal growth, the positions of the concepti are more variable and their movements significantly increase, making the measurement less accurate. In this regard, the measurement of the largest transverse diameter between both parietal bones, evaluated as the biparietal diameter (BPD), shows greater reliability. Previous studies by ultrasound showed that the BPD is the most representative parameter of the gestational age during the second third of pregnancy in sheep [25]. The strong correlation of BPD with the gestational age shown in our study confirms this parameter as a reliable indicator of fetal age, as previously observed in other domestic ungulates such as pig [16], sheep [26,27] and cattle [28]. At this stage of development, in addition to the use of the BPD, the search and observation of specific anatomical structures could be advisable. In this respect, the development of the skeleton is of great importance.

In the last years, various studies tried to investigate the dynamics of the development of the conceptus in mammals and in sheep, several studies focused on the skeletal development, since this species is considered an excellent model for human(s) [5,8]. In the past, most of the studies used radiology to describe skeletal development in ruminants, pigs, cats and humans [29,30,31,32,33,34]. The spread of the use of ultrasound has allowed a change in the investigation approaches. Ultrasonography is a well-validated technique and largely used to detect mineralization and to measure fetal bones and ossification centers [35,36]. However, even if significant progress has been made, the precise detection of skeletal ossification loci and dynamics needs to be confirmed by other estimation methods.

Our study represents an innovative attempt to describe fetal skeleton development during the first part of gestation in sheep using ultrasonography associated with a differential staining technique that allows the observation of the first ossification processes. The first step was to adapt the staining and clarification protocols to the ovine fetuses, especially the fixation times and methods. Indeed, the conceptus must be fixed exclusively in ethanol and not in formalin, to avoid non-specific staining of embryonic structures. Furthermore, embryos with an age of less than 30 days cannot be processed due to the richness of glycosaminoglycans of the young connective tissue that interfere with the staining of the cartilage matrix. As a result, we obtained an overview of osteochondral temporal dynamics in the early stage of development from 30 days up to 70 days. In effect, while a previous study described single skeletal components [37], no research has previously reported data on the complete skeleton in sheep with this technique.

The first evidence of ossification (bone mineralization) in our concepti was observed in the skull towards the 40th day of gestation (26.6% of the total gestational period (GP)) starting from the mandible, earlier compared to that described in sheep by Harrys in 1937, but similar to observations in domestic pig (37 days of gestation, 26% of GP; [16], cow (25% of GP; [38,39]) humans (26% of GP; [40]) and some wild mammals such as lowland paca (28% of GP; [41]), collared and white-lipped peccary [42] and African elephant [43]. The development of the forelimbs and hind limbs was evidenced by the early appearance of ossification (40 and 45 days of gestation, 26,6% and 30% of GP, respectively) like other precocial animals (cow [38], African elephant [43], collared and white-lipped peccary [42]), contrary to what happens in altricial animals as rabbit [44], rats [45], domestic carnivores [9], and primates such as marmosets [46], where the first mineralization of the appendicular skeleton was identified at 66%, 75%, 72.6% and 70% of GP, respectively.

In mammals, except for humans, the gestation period seems to be correlated with the size of the offspring and its competence (altricial vs. precocial; [47]); furthermore, there seems to be a correspondence between the length of gestation and the timing of ossification [43]. Sheep, as a mammal with a long gestation, is precocial and begins ossification earlier than other mammalian species with a long gestation and altricial offspring. In accordance, our data on osteo–chondral dynamics have shown fetuses with the ossification process extended to the whole skeleton already at 70 days of pregnancy, an early phase of gestation equal to 46.6% of GP. The timing of ossification and the growth rate of skeletal components are very important to assess the normal prenatal growth, as indicated in different species (humans [30], pigs [48], dogs [49], mice [45]). The correct bone development provides an adequate structural support to the whole organism and its locomotive function [50]; therefore, adequate skeletal development during gestation is essential for the good performance of the offspring after birth, which has implications for the survival rate of lambs [51]. The study of the dynamics of ossification of the limbs provides a great help in estimating the age. Especially in the early period of pregnancy, when in the ovine species the bone growth is very fast, this knowledge may be useful for gestational management and early diagnosis of skeletal diseases.

## 5. Conclusions

Our study using staining and clarification techniques combined with systematic ultrasound has confirmed that biparietal diameter is the most accurate parameter for estimation of gestational age in sheep. Furthermore, our study suggests that the ossification of the tibia, due to its growth characteristics and its particular shape, could be considered a valid parameter to estimate fetal age by ultrasound. To our knowledge, this study is the first observation on the overall skeletal development of the ovine species.

## Figures and Tables

**Table 1:** Dynamics of ossification during fetal growth between day 30 and 70 of gestation in sheep.

CRL (cm)	BPD (cm)	Day of Pregnancy (% of Gestational Period)	Dynamic of Ossification
2.01 ± 0.045– 2.54 ± 0.022	0.49 ± 0.002– 0.66 ± 0.007	30th–35th (20–23)	Skeleton cartilaginous
3.34 ± 0.088– 4.05 ± 0.058	1.03 ± 0.01– 1.12 ± 0.026	40th–45th (26.6–30)	Beginning of jaw ossification and humerus and radius diaphysis
7.06 ± 0.037– 8.15 ± 1.32	1.38 ± 0.026– 1.63 ± 0.055	50th–55th (33.3–36.6)	Beginning of ossification of rachis, ossification of ribs, diaphysis of femur, and tibia. Beginning of intra-membranous ossification of the skull.
8.65 ± 0.058– 9.2 ± 0.153	2.41 ± 0.024– 2.52 ± 0.03	65th–70th (43.3–46.6)	Almost all skeleton is ossified, except ends of the limbs, cervical vertebra and chondrocostal junctions.

## Data Availability

The data produced during the current study are available from the corresponding author on reasonable request.

## Supplementary Materials

The following supporting information can be downloaded at: https://www.mdpi.com/article/10.3390/ani13050773/s1, Table S1: Table 1. Values of Crown Rump Length (CRL) expressed as mean ± SEM, measured by ultrasonography (eco) and directly by a ruler (vivo) in ovine conceptus from day 30 to day 70 of pregnancy; Table S2: Values of Biparietal Diameter (BPD) in cm expressed as mean ± SEM, measured by ultrasonography (eco) in ovine conceptus from day 30 to day 70 of pregnancy.
