# Supplementary material for: Fetal Growth and Osteogenesis Dynamics during Early Development in the Ovine Species
Source: Animals (Basel). 2023 Feb 21;13(5):773. doi: 10.3390/ani13050773 (PMC10000038; doi:10.3390/ani13050773)
Supplement: Supplementary file 1 [file animals-13-00773-s001.zip › animals-2167032-supplementary.pdf]

Supplementary files

Table S1: Values of Crown Rump Length (CRL) expressed as mean ±SEM, measured by ultrasonography (eco) and directly by a ruler (vivo) in ovine conceptus from day 30 to day 70 of pregnancy. N= number of measured samples for each gestational age.

Day of pregnancy	CRL (cm) in eco group			CRL (cm) in vivo group		
	Mean	SEM	N	Mean	SEM	N
22th	0.986	±0.021	3	0.997	±0.071	3
24th	1.175	±0.005	2	1.15	±0.050	2
25th	1.24	±0.012	3	1.37	±0.033	3
28th	1.70	±0.030	3	1.87	±0.120	3
30th	2.01	±0.045	4	2.16	±0.057	4
32th	2.33	±0.029	3	2.40	±0.058	3
35th	2.54	±0.022	4	2.62	±0.031	4
40th	3.34	±0.088	4	3.57	±0.051	4
42th	3.67	±0.040	2	3.70	±0.10	2
45th	4.05	±0.058	3	4.27	±0.082	3
50th	7.06	±0.037	3	7.35	±0.058	3
55th	8.15	±0.132	4	8.20	±0.041	4
60th	8.23	±0.033	3	8.57	±0.067	3
65th	8.65	±0.058	3	8.85	±0.088	3
70th	9.2	±0.153	2	9.27	±0.088	2

Table S2: Values of Biparietal Diameter (BPD) in cm expressed as mean ±SEM, measured by ultrasonography (eco) in ovine conceptus from day 30 to day 70 of pregnancy. N= number of measured samples for each gestational age.

Day of pregnancy	BPD (cm)		
	mean	SE	N
30th	0.499	±0.0015	2
35th	0.669	±0.005	2
40th	1.03	±0.010	2
45th	1.123	±0.026	3
50th	1.38	±0.0265	4
55th	1.63	±0.055	4
60th	1.96	±0.0285	3
65th	2.41	±0.0240	3
70th	2.52	±0.03	2
